# Association of TCF7L2 Genetic Polymorphisms with Type 2 Diabetes Mellitus in the Uygur Population of China

**DOI:** 10.3390/ijerph120911797

**Published:** 2015-09-18

**Authors:** Hua Yao, Zhiqiang Wang, Tingting Wang, Yan Ma, Yinxia Su, Qi Ma, Li Wang, Jun Zhu

**Affiliations:** 1 Xinjiang Key Laboratory of Metabolic Disease Research, The First Affiliated Hospital of Xinjiang Medical University, Urumqi, Xinjiang 830054, China; E-Mails: maqi1212@126.com (Q.M.); wl78154189@sina.cn (L.W.); zhujun6677@163.com (J.Z.); 2 Department of Public Health, Xinjiang Medical University, Urumqi, Xinjiang 830011, China; E-Mails: zhiqiangwang1987@sina.com (Z.W.); wangtingting2013@sina.com (T.W.); mayan16@sina.com (Y.M.); yinxia_su@163.com (Y.S.)

**Keywords:** TCF7L2, Single nucleotide polymorphism, Type 2 diabetes mellitus, Case–control study

## Abstract

Background: Genetic polymorphisms of the transcription factor 7-like 2 (TCF7L2) gene have been reported to be strongly associated with type 2 diabetes mellitus (T2DM) in Icelandic, Danish and American populations and further replicated in other European populations, African Americans, Mexican Americans, and Asian populations. The aim of the present study was to investigate the association of TCF7L2 gene polymorphisms with T2DM in a Uygur population of China. Methods: 877 T2DM patients and 871 controls were selected for the present study. Two single nucleotide polymorphisms (SNPs) (rs12255372 and rs7901695) were genotyped by using matrix-assisted laser desorption/ionization time-of-flight (MALDI-TOF) mass spectrometry. The associations of SNPs and haplotypes with T2DM and linkage disequilibrium (LD) structure of the TCF7L2 gene were analyzed. Results: For total participants and male, the distribution of rs12255372 alleles and the dominant model (Guanine Guanine (GG) genotype *vs.* Guanine Thymine (GT) genotype + Thymine Thymine (TT) genotype) showed significant difference between T2DM and control subjects (for allele: *p* = 0.013 and *p* = 0.002, respectively; for dominant model: *p* = 0.028 and *p* = 0.008, respectively). The distribution of rs7901695 alleles and the dominant model (TT genotype *vs.* Thymine Cytosine (TC) genotype + Cytosine Cytosine (CC) genotype) for total participants and male showed significant difference between T2DM and control subjects (for allele: both *p* = 0.001; for dominant model: *p* = 0.006 and *p* = 0.008, respectively). Conclusions: Our data suggested that the genetic polymorphisms of the TCF7L2 gene were associated with T2DM in the Uygur population of China.

## 1. Introduction

Type 2 diabetes mellitus (T2DM) is a complex metabolic disease characterized by chronic hyperglycemia resulting from the combination of genetic and environmental factors [1]. Most individuals with T2DM suffer various complications, such as coronary heart disease, diabetic nephropathy, neuropathy and retinopathy. Recent years, several genetic variants have shown association with T2DM among different ethnic populations. These include the Pro12Ala polymorphism in the peroxisome proliferator activator receptor gamma (PPARG) gene [2], the E23K polymorphism in the potassium inwardly rectifying channel, subfamily J, member 11(KCNJ11) gene [3], and the genetic variants of the calpain 10 (CAPN10) gene [4] and the high mobility group AT-hook 1(HMGA1) gene [5]. In 2006, Grant *et al.* [6] identified one microsatellite DG10S478 within intron 3 of the transcription factor 7-like 2 gene (TCF7L2; formerly TCF4) was associated with type 2 diabetes (*p* = 2.1 × 10^−9^) in Icelandic individuals. This association was replicated in a Danish cohort (*p* = 4.8 × 10^−3^) and in a US cohort (*p* = 3.3 × 10^−9^). The authors then genotyped the five single nucleotide polymorphisms (SNPs) (rs12255372, rs7903146, rs7901695, rs11196205, and rs7895340) within a well-defined linkage disequilibrium (LD) block of 92.1 kb spanning intron 3 and intron 4 of TCF7L2 and showed association between all five SNPs and T2DM in all three cohorts. Further studies in other European populations [6,7,8,9,10,11], Mexican Americans [12], African Americans [13], and Asian populations [14,15,16,17,18] identified the strong association with an estimated population attributable risk of 17–28%. However, the results previously reported in Chinese Han population are often inconsistent [16,17,18]. The TCF7L2 gene product is a high mobility group box-containing transcription factor that plays an important role in the Wnt signaling pathway. This pathway is a key component to the regulation of cell proliferation and differentiation [19,20]. The Xinjiang Uygur Autonomous Region of China is part of the ancient Silk Road and borders eight countries including Russia, Kazakhstan, Kyrgyzstan, Tajikistan, Pakistan, Mongolia, India, and Afghanistan. There are more than 13 ethnic groups living in this area. Among them, the Uygur people account for 46%, and Han account for 40% [21]. In spite of the presence of these people in the same geographic area, each ethnic group is relatively distinct with little intermingling. The Uygur people originate from inter-marriage between Caucasians and Mongolians, and have their own language, culture, genetic background, lifestyle, and dietary habits [22]. However, the association study of the genetic polymorphism of TCF7L2 with T2DM in a Uygur population of China has been lacking.

In the present study, we aimed to investigate the association of TCF7L2 gene polymorphisms with T2DM in a Uygur population of China.

## 2. Material and Methods

### 2.1. Ethical Approval of the Study Protocol

The present study was conducted in accordance with the Declaration of Helsinki guidelines, and written informed consent was obtained from each individual according to a protocol approved by the Ethics Committee of the First Affiliated Hospital of Xinjiang Medical University.

### 2.2. Subjects

A total of 1748 Uygur Chinese subjects (1104 male, 644 female) were enrolled in the current study from Xinjiang Uygur Autonomous Region of China. Among them, 877 subjects diagnosed with T2DM at the inpatient department of First Affiliated Hospital of Xinjiang Medical University from January 2012 to December 2013 were recruited. We defined diabetes by using the American Diabetes Association (ADA) 2009 criteria [23] (fasting plasma glucose ≥7.0 mmol/L (≥126 mg/dL)) or self-reported current diabetes treatments in the survey. For each T2DM patient group, we selected healthy participants matched for ethnicity, sex and age as the controls. Controls with a fasting plasma glucose (FPG) concentration of <6.1 mmol/L and a 2-h plasma glucose of <7.8 mmol/L were enrolled from the center of health examination at the same hospital, these participants had no family history of T2DM. Demographic and anthropometric characteristics were collected by interviewer-administered questionnaire. Information on demographic characteristics including age, gender, ethnicity, education, address, occupation, and household income were collected from all participants. Systemic arterial hypertension was defined as a systolic blood pressure of ≥140 mmHg and/or a diastolic blood pressure of ≥90 mmHg [24], on at least two separate occasions, or anti-hypertensive treatment. Hypercholesterolaemia was defined as a documented total cholesterol value ≥240 mg/dl (≥6.2mmol/L) or current treatment with cholesterol-lowering medication. All participants underwent a standardized physical examination performed by experienced research staff. Anthropometric measurements were conducted in light clothing and without shoes. Height was measured to the nearest 0.1 cm, and weight was measured with a standard scale in the upright position to the nearest 0.1 kg. Body mass index (BMI) was calculated as weight (kg) divided by height squared (m^2^). Sitting blood pressure was measured three times during 10 min, and the median value was used for statistical analysis. Subjects with impaired renal function, malignancy, connective-tissue disease, or chronic inflammatory disease were excluded from the study.

### 2.3. Biochemical Analysis

Fasting blood samples (fasting at least 8 h) were taken from all participants. All of the collected blood samples were transported on dry ice at prearranged intervals to laboratory. The blood samples were collected into plain tubes and ethylene diamine tetraacetic acid (EDTA) tubes. Plain vacutainer consist of the serum sample was used to analyze the biochemical parameters and the EDTA sample was used for molecular analysis. The blood samples were centrifuged at 4000 × g for 5 min to separate the plasma content. Genomic DNA was extracted from the peripheral blood leukocytes using a DNA extraction Kit (Beijing Biotech Company Limited, Beijing, China). The DNA samples were stored at −80 °C until use. When used, the DNA was diluted to 50 ng/μL concentration. We measured the serum concentration of total cholesterol (TC), triglyceride (TG), high-density lipoprotein cholesterol (HDL-C), low-density lipoprotein cholesterol (LDL-C), blood urea nitrogen (BUN), creatinine (Cr) and fasting glucose using equipment for chemical analysis (Dimension AR/AVL Clinical Chemistry System, Newark, NJ, USA) employed by the Clinical Laboratory Department of the First Affiliated Hospital of Xinjiang Medical University.

### 2.4. Genotyping of TCF7L2 Gene

There are 5501 SNPs for the human TCF7L2 gene listed in the National Center for Biotechnology Information SNP database [25]. We screened the data for the Tag SNPs on the International HapMap Project website [26]. Using the Haploview 4.2 software (Cambridge, MA, USA) [27] and the HapMap phrase II database, we obtained two tagging SNPs (rs12255372 and rs7901695) by using minor allele frequency (MAF) ≥0.05 and linkage disequilibrium patterns with r^2^ ≥ 0.8 as a cutoff. In addition, the rs12255372 and rs7901695 of TCF7L2 gene were selected in this study on the basis of published studies [7,8,9,10,11,12,13,14,15,16,17,18,19,20]. Genotyping in this present case-control study was confirmed by MALDI-TOF Mass Spectrometry. The amplification polymerase chain reaction (PCR) primer pairs and the extend primers were designed using MassARRAY Assay Design software version 3.1 (Table 1). PCR reactions were performed in standard 384-well plates containing 10 ng genomic DNA, 0.5 U of Taq polymerase (Qiagen, Hilden, Germany), 500 μmol of deoxyribonucleoside triphosphates (dNTPs) and 100 nmol of both forward and reverse PCR primers. Thermocycling conditions consisted of an initial 15 min denaturation at 94 °C, followed by 45 cycles of 20 s denaturing at 94 °C, 30 s annealing at 56 °C and 60 s extension at 72 °C. PCR products were purified by incubation at 37 °C using 2 μL of shrimp alkaline phosphatase for 40 min followed by a 5 min inactivation at 85 °C. A primer extension reaction mixture including 0.2 μL of termination mix, 0.041 μL of DNA polymerase and 0.804 μL of the extension primers was used in both the initial (denaturation at 94 °C for 30 s, followed by 5 annealing and extension cycles at 52 °C and 80 °C, respectively) and secondary (40 cycles of 5 s denaturation at 94 °C, 5 s annealing at 52 °C and 5 s extension at 80 °C) iPLEX reactions. A final extension for 3 min at 72 °C was conducted prior to cooling at 4 °C. Products were diluted and desalted with 16 μL sterile water and 6 mg of clean resin prior to spotting onto a SpectroChip for analysis in the Compact Mass Spectrometer (Sequenom, San Diego, California, USA), using MassARRAY Typer software version 4.0 (Sequenom, San Diego, California, USA). The accuracy of the genotyping was determined by assessing the genotype concordance between duplicate samples. We obtained a 100% concordance between the genotyped duplicate samples for the single nucleotide polymorphism (SNP). The genotyping success rate was 100%.

### 2.5. Statistical Analysis

All statistical analysis was performed by using Statistical Package for the Social Sciences (SPSS17.0, Chicago, USA). All continuous variables were expressed as the mean ± standard deviation (SD). The differences between the T2DM patients and the control participants were assessed using independent samples t test. Differences in enumeration data between T2DM patients and control participants were analyzed using the *x^2^* test. Categorical variables such as allele and genotype frequencies among T2DM cases and controls were compared using the *x^2^* test. Hardy-Weinberg equilibrium was assessed by *x^2^* analysis. Haplotype analysis and linkage disequilibrium coefficients were calculated by use of Haploview 4.2 (Cambridge, MA, USA) [27]. Logistic regression analysis with effect ratios (odds ratio (*OR*) and 95% confidence interval (*CI*)) were used to assess contribution of major risk factors. *p* value < 0.05 was considered statistically significant.

## 3. Results

Table 2 shows demographic and clinical characteristics of T2DM patients and control subjects in the Uygur population. For total participants, BMI, systolic blood pressure (SBP), the serum concentration of glucose, TC, and BUN were significantly higher for T2DM patients than for control subjects (*p* < 0.05), whereas the serum concentration of HDL-C, LDL-C, and uric acid (UA) were significantly lower for T2DM patients than for control subjects (*p* < 0.05). For total participants, there was no significant difference in the following variables between T2DM patients and control subjects: age, diastolic blood pressure (DBP), the serum concentration of TG, creatinine (all *p* > 0.05). For male participants, BMI, the serum concentration of glucose, TC, and BUN were significantly higher for T2DM patients than for control subjects (*p* < 0.05), whereas DBP, the serum concentration of HDL-C, UA were significantly lower for T2DM patients than for control subjects (*p* < 0.05). For male participants, there was no significant difference in the following variables between T2DM patients and control subjects: age, SBP, the serum concentration of TG, LDL-C, creatinine (all *p* > 0.05). For female participants, SBP, the serum concentration of glucose, total cholesterol (TC) were significantly higher for T2DM patients than for control subjects (*p* < 0.05), whereas the serum concentration of HDL-C were significantly lower for T2DM patients than for control subjects (*p* < 0.05). For female participants, there was no significant difference in the following variables between T2DM patients and control subjects: age, BMI, DBP, the serum concentration of TG, LDL-C, UA, creatinine, BUN (all *p* > 0.05).

Table 3 shows the distribution of the genotypes and alleles for the two SNPs of TCF7L2 gene in the Uygur population. The genotypic distribution for each of the SNPs was in agreement with the predicted Hardy-Weinberg equilibrium values (*p* > 0.05 in the T2DM and control groups, data not shown).

**Table 1:** Primer sequences of each SNP.

SNPs	PCR Primers Sequences	Amplicon Size (bp)	Temperature (°C)	Guanine Cytosine (GC) (%)
	Forward primer:			
	ACGTTGGATGCAGAGGCCTGAGTAATTATC			
rs12255372		101	47.3	42.1
	Reverse primer:			
	ACGTTGGATGTGCAAATCCAGCAGGTTAGC			
	Forward primer:			
	ACGTTGGATGTGTGGATTTGCCTGTTCTTG			
rs7901695		117	47	50
	Reverse primer:			
	ACGTTGGATGCTTGAGAACCGTATGCTAAG			

**Table 2:** Demographic and clinical characteristics of study participants.

Characteristics	Total			Male			Female		
	T2DM	Control	*p* value	T2DM	Control	*p* value	T2DM	Control	*p* value
Number (n)	877	871		542	562		335	309	
age (years)	51.14 ± 9.66	50.34 ± 9.70	0.065	51.07 ± 9.79	50.42 ± 9.85	0.237	51.24 ± 9.45	50.20 ± 9.45	0.133
BMI (kg/m^2^)	27.59 ± 4.26	26.96 ± 3.96	0.016 *	27.63 ± 3.85	26.96 ± 3.66	0.025 *	27.52 ± 4.87	26.95 ± 4.49	0.259
SBP (mmHg)	126.86 ± 18.92	122.72 ± 17.48	0.009 *	125.23 ± 16.34	123.85 ± 18.69	0.465	129.51 ± 22.26	120.90 ± 15.34	<0.001^*^
DBP (mmHg)	79.33 ± 11.88	81.36 ± 13.93	0.062	78.87 ± 10.81	82.91 ± 14.98	0.002 *	80.08 ± 13.43	78.78 ± 11.64	0.494
Glu (mmol/L)	9.57 ± 3.43	5.01 ± 0.91	<0.001 *	9.60 ± 3.36	4.99 ± 0.87	<0.001 *	9.52 ± 3.55	5.04 ± 0.99	<0.001^*^
TG (mmol/L)	2.43 ± 2.17	2.43 ± 2.10	0.946	2.68 ± 2.50	2.65 ± 2.27	0.868	2.01 ± 1.38	2.06 ± 1.69	0.676
TC (mmol/L)	4.66 ± 1.36	4.33 ± 1.68	<0.001 *	4.63 ± 1.43	4.26 ± 1.66	<0.001 *	4.72 ± 1.23	4.44 ± 1.71	0.017^*^
HDL (mmol/L)	0.96 ± 0.33	1.25 ± 0.33	<0.001 *	0.91 ± 0.28	1.18 ± 0.30	<0.001 *	1.04 ± 0.40	1.37 ± 0.33	<0.001^*^
LDL (mmol/L)	2.87 ± 1.46	3.01 ± 0.82	0.017 *	2.87 ± 1.72	3.01 ± 0.79	0.088	2.87 ± 0.84	3.00 ± 0.88	0.056
UA (mmol/L)	268.68 ± 84.12	282.38 ± 70.40	<0.001 *	286.86 ± 85.48	307.11 ± 61.45	<0.001 *	238.32 ± 72.35	240.59 ± 64.64	0.662
Cr (umol/L)	68.66 ± 43.62	71.11 ± 17.88	0.124	75.16 ± 44.69	76.27 ± 13.84	0.577	57.71 ± 39.48	62.53 ± 20.38	0.051
BUN (mmol/L)	5.20 ± 2.29	4.96 ± 1.44	0.007 *	5.42 ± 2.34	5.08 ± 1.35	0.003 *	4.83 ± 2.16	4.76 ± 1.55	0.631

Continuous variables are expressed as mean ± SD. Categorical variables are expressed as percentages. T2DM: type 2 diabetes mellitus; BMI: body mass index; SBP: systolic blood pressure; DBP: diastolic blood pressure; Glu: glucose; TG: triglyceride; TC: total cholesterol; HDL: high density lipoprotein; LDL: low density lipoprotein; UA: uric acid; Cr: creatinine; BUN: blood urea nitrogen. The *p* value of the continuous variables was calculated by the Independent t-test. The *p* value of the categorical variables was calculated by X^2^ test. * *p* < 0.05.

**Table 3:** Genotype and allele distributions in patients with T2DM and control subjects.

Variants	Total			Male			Female		
	T2DM *n* (%)	Control *n* (%)	*p* value	T2DM *n* (%)	Control *n* (%)	*p* value	T2DM *n* (%)	Control *n* (%)	*p* value
rs12255372									
Genotyping									
GG	572 (65.2)	611 (70.2)		344 (63.5)	399 (71.0)		228 (68.1)	212 (68.6)	
GT	270 (30.8)	238 (27.3)		172 (31.7)	152 (27.0)		98 (29.2)	86 (27.8)	
TT	35 (4.0)	22 (2.5)	0.044 *	26 (4.8)	11 (2.0)	0.004 *	9 (2.7)	11 (3.6)	0.773
Dominant model									
GG	572 (65.2)	611 (70.2)		344 (63.5)	399 (71.0)		228 (68.1)	212 (68.6)	
GT+TT	305 (34.8)	260 (29.8)	0.028 *	198 (36.5)	163 (29.0)	0.008 *	107 (31.9)	97 (31.4)	0.881
Recessive model									
TT	35 (4.0)	22 (2.5)		26 (4.8)	11 (2.0)		9 (2.7)	11 (3.6)	
GG+GT	842(96.0)	849 (97.5)	0.085	516 (95.2)	551 (98.0)	0.009 *	326 (97.3)	298 (96.4)	0.523
Additive model									
GG	572 (65.2)	611 (70.2)		344 (63.5)	399 (71.0)		228 (68.1)	212 (68.6)	
GT	270 (30.8)	238 (27.3)		172 (31.7)	152 (27.0)		98 (29.2)	86 (27.8)	
TT	35 (4.0)	22 (2.5)	0.014 *	26 (4.8)	11 (2.0)	0.002 *	9 (2.7)	11 (3.6)	0.939
Allele									
G	1414 (80.6)	1460 (83.8)		860 (79.3)	950 (84.5)		554 (82.7)	510 (82.5)	
T	340 (19.4)	282 (16.2)	0.013 *	224 (20.7)	174 (15.5)	0.002 *	116 (17.3)	108 (17.5)	0.939
rs7901695									
Genotyping									
TT	535 (61.0)	586 (67.3)		325 (60.0)	380 (67.6)		210 (62.7)	206 (66.7)	
TC	295 (33.6)	262 (30.1)		184 (33.9)	171 (30.4)		111 (33.1)	91 (29.4)	
CC	47 (5.4)	23 (2.6)	0.002 *	33 (6.1)	11 (2.0)	<0.001 *	14 (4.2)	12 (3.9)	0.570
Dominant model									
TT	535 (61.0)	586 (67.3)		325 (60.0)	380 (67.6)		210 (62.7)	206 (66.7)	
TC+CC	342 (39.0)	285 (32.7)	0.006 *	217 (40.0)	182 (32.4)	0.008 *	125 (37.3)	103 (33.3)	0.291
Recessive model									
CC	47 (5.4)	23 (2.6)		33 (6.1)	11 (2.0)		14 (4.2)	12 (3.9)	
TT+TC	830 (94.6)	848 (97.4)	0.004 *	509 (93.9)	551 (98.0)	<0.001 *	321 (95.8)	297 (96.1)	0.849
Additive model									
TT	535 (61.0)	586 (67.3)		325 (60.0)	380 (67.6)		210 (62.7)	206 (66.7)	
TC	295 (33.6)	262 (30.1)		184 (33.9)	171 (30.4)		111 (33.1)	91 (29.4)	
CC	47 (5.4)	23 (2.6)	0.001 *	33 (6.1)	11 (2.0)	0.001 *	14 (4.2)	12 (3.9)	0.338
Allele									
T	1365 (77.8)	1434 (82.3)		834 (76.9)	931 (82.8)		531 (79.3)	503 (81.4)	
C	389 (22.2)	308 (17.7)	0.001 *	250 (23.1)	193 (17.2)	0.001 *	139 (20.7)	115 (18.6)	0.335

* *p* <0.05.

For total participants, distribution of rs12255372 genotypes, dominant model (GG *vs.* GT + TT), additive model (GG *vs.* GT *vs.* TT) and allele frequency showed significant difference between T2DM and control subjects (*p* = 0.044, *p* = 0.028, *p* = 0.014, *p* = 0.013). For male, distribution of rs12255372 genotypes, dominant model (GG *vs.* GT + TT), recessive model (TT *vs.* GG + GT), additive model (GG *vs.* GT *vs.* TT) and allele frequency showed significant difference between T2DM and control subjects (*p* = 0.004, *p* = 0.008, *p* = 0.009, *p* = 0.002, *p* = 0.002). In the present study, when male and female were analyzed separately, for total participants and male, T allele of rs12255372 was significantly higher in subjects with T2DM than in controls (total: 19.4% *vs.* 16.2%; male: 20.7% *vs.* 15.5%), the dominant model (GG *vs.* GT + TT) of rs12255372 was significantly higher in controls than in subjects with T2DM (total: 70.2% *vs.* 65.2%; male: 71.0% *vs.* 63.5%). For male, the recessive model (TT *vs.* GG + GT) of rs12255372 was significantly higher in subjects with T2DM than in controls (4.8% *vs.* 2.0%). There was no significant difference between T2DM and control subjects in female for distribution of rs12255372 genotypes, dominant model (GG *vs.* GT + TT), recessive model (TT *vs.* GG + GT), additive model (GG *vs.* GT *vs.* TT) and allelic distribution (*p* > 0.05).

For total participants, distribution of rs7901695 genotypes, dominant model (TT *vs.* TC + CC), recessive model (CC *vs.* TT + TC), additive model (TT *vs.* TC *vs.* CC) and allele frequency showed significant difference between T2DM and control subjects (*p* = 0.002, *p* = 0.006, *p* = 0.004, *p* = 0.001, *p* = 0.001). For male, distribution of rs7901695 genotypes, dominant model (TT *vs.* TC + CC), recessive model (CC *vs.* TT + TC), additive model (TT *vs.* TC *vs.* CC) and allele frequency showed significant difference between T2DM and control subjects (*p* < 0.001, *p* = 0.008, *p* < 0.001, *p* = 0.001, *p* = 0.001). For total participants and male, Cytosine (C) allele of rs7901695 was significantly higher in subjects with T2DM than in controls (total: 22.2% *vs.* 17.7%; male: 23.1% *vs.* 17.2%), the dominant model (TT *vs.* TC + CC) of rs7901695 was significantly higher in controls than in subjects with T2DM (total: 67.3% *vs.* 61.0%; male: 67.6% *vs.* 60.0%), the recessive model (CC *vs.* TT + TC) of rs7901695 was significantly higher in subjects with T2DM than in controls (total: 5.4% *vs.* 2.6%; male: 6.1% *vs.* 2.0%). There was no significant difference between T2DM and control subjects in female for distribution of rs7901695 genotypes, dominant model (TT *vs.* TC + CC), recessive model (CC *vs.* TT + TC), additive model (TT *vs.* TC *vs.* CC) and allelic distribution (*p* > 0.05).

Table 4 shows multivariable logistic regression analysis combining genotypes with following variables: the serum concentration of TG, TC, HDL-C, and LDL-C, which were the major confounding factors for T2DM. In the present study, when male and female were analyzed separately, for total participants and male, after multivariate adjustment, rs12255372 remain significantly associated with T2DM in the dominant model (total: *OR* = 1.301, 95% confidence interval (*CI*): 1.019–1.660, *p* = 0.035; male: *OR* = 1.475, 95% confidence interval (*CI*): 1.080–2.013, *p* = 0.014) and in the additive model (total: *OR* = 1.280, 95% confidence interval (*CI*): 1.039–1.577, *p* = 0.021; male: *OR* = 1.467, 95% confidence interval (*CI*): 1.123–1.914, *p* = 0.005; data not shown for the additive model). For male, after multivariate adjustment, rs12255372 remained significantly associated with T2DM in the recessive model (*OR* = 2.477, 95% confidence interval (*CI*): 1.089–5.632, *p* = 0.030; data not shown for the recessive model).

For total participant and male (Table 5), after multivariate adjustment, rs7901695 remain significantly associated with T2DM in the dominant model (total: *OR* = 1.418, 95% confidence interval (*CI*): 1.117–1.801, *p* = 0.004; male: *OR* = 1.526, 95% confidence interval (*CI*): 1.126–2.067, *p* = 0.006), in the recessive model (total: *OR* = 2.417, 95% confidence interval (*CI)*: 1.341–4.356, *p* = 0.003; male: *OR* = 3.825, 95% confidence interval (*CI*): 1.721–8.498, *p* = 0.001; data not shown for the recessive model) and in the additive model (total: *OR* = 1.427, 95% confidence interval (*CI*): 1.166–1.746, *p* = 0.001; male: *OR* = 1.581, 95% confidence interval (*CI*): 1.222–2.045, *p* < 0.001; data not shown for the additive model).

In the haplotype-based case-control analysis, haplotypes were established in two representative common SNPs (Table 6). The two SNPs, rs12255372 and rs7901695, were found to be in strong linkage disequilibrium with each other (*D’* = 0.879, *r^2^* = 0.671) and therefore formed a haplotype block. For total participants and male, the frequency of C-T haplotype was significantly higher in subjects with T2DM than in controls (total: *p* = 0.006; male: *p* < 0.001), whereas the frequency of T-G haplotype was significantly higher in controls than in subjects with T2DM (both *p* = 0.002). For female, the frequency of Cytosine–Guanine (C–G) haplotype was significantly higher in subjects with T2DM than in controls (*p* = 0.044).

**Table 4:** Multiple logistic regression analysis for T2DM patients and control subjects (rs12255372).

Risk Factors	Total			Male			Female		
	*OR*	95% *CI*	*p*	*OR*	95% *CI*	*p*	*OR*	95% *CI*	*p*
dominant model (GG *vs.* GT + TT)	1.301	1.019–1.660	0.035 *	1.475	1.080–2.013	0.014 *	1.048	0.699–1.571	0.819
TG	0.876	0.822–0.932	<0.001 *	0.880	0.819–0.945	<0.001 *	0.927	0.793–1.084	0.345
TC	1.509	1.338–1.701	<0.001 *	1.467	1.285–1.675	<0.001 *	1.643	1.277–2.114	<0.001 *
HDL	0.029	0.018–0.045	<0.001 *	0.015	0.008–0.030	<0.001 *	0.034	0.017–0.069	<0.001 *
LDL	0.779	0.646–0.940	0.009 *	0.863	0.712–1.046	0.134	0.657	0.448–0.964	0.032 *

T2DM: type 2 diabetes mellitus; TG: triglyceride; TC: total cholesterol; HDL: high density lipoprotein; LDL: low density lipoprotein;* *p* < 0.05.

**Table 5:** Multiple logistic regression analysis for T2DM patients and control subjects (rs7901695).

Risk Factors	Total			Male			Female		
	*OR*	95% *CI*	*p*	*OR*	95% *CI*	*p*	*OR*	95% *CI*	*p*
dominant model (TT *vs.* TC + CC)	1.418	1.117–1.801	0.004 *	1.526	1.126–2.067	0.006 *	1.281	0.860–1.910	0.223
TG	0.876	0.823–0.933	<0.001 *	0.881	0.821–0.947	0.001 *	0.928	0.793–1.087	0.353
TC	1.512	1.340–1.705	<0.001 *	1.469	1.287–1.677	<0.001 *	1.655	1.284–2.134	<0.001 *
HDL	0.028	0.018–0.045	<0.001 *	0.015	0.008–0.029	<0.001 *	0.034	0.017–0.068	<0.001 *
LDL	0.782	0.648–0.943	0.010 *	0.869	0.721–1.047	0.140	0.648	0.441–0.952	0.027 *

T2DM: type 2 diabetes mellitus; TG: triglyceride; TC: total cholesterol; HDL: high density lipoprotein; LDL: low density lipoprotein;* *p* < 0.05.

**Table 6:** Haplotype analysis in patients with T2DM and control subjects.

Variables	Haplotype		Total			Male			Female		
	rs7901695	rs12255372	T2DM (%)	Control (%)	*p*	T2DM (%)	Control (%)	*p*	T2DM (%)	Control (%)	*p*
H1	T	G	76.2	80.5	0.002 *	75.6	81.1	0.002 *	77.2	79.4	0.353
H2	C	T	17.8	14.3	0.006 *	19.3	13.7	<0.001 *	15.3	15.4	0.936
H3	C	G	4.4	3.3	0.102	3.8	3.4	0.688	5.5	3.2	0.044 *
H4	T	T	1.6	1.8	0.596	1.4	1.7	0.459	2.0	2.0	0.998

T2DM: type 2 diabetes mellitus; * *p* < 0.05.

## 4. Discussion

We found that variations in the TCF7L2 gene were associated with T2DM in a Uygur population of China. After multivariate adjustment, the associations between the TCF7L2 gene polymorphisms with T2DM were not modified. To the best of our knowledge, this was the first study to investigate the association of the TCF7L2 gene polymorphisms with T2DM in a Uygur population of China.

The previous studies have focused on the important role of TCF7L2 in oncogenesis and cancer progression [28,29,30,31]. Functional analyses are required to identify the role of TCF7L2 in T2DM and to determine how variants of this gene affect susceptibility to T2DM. T2DM is characterized by impaired insulin secretion in response to increased metabolic demand. This can be attributed to defective beta-cell mass and/or impaired beta-cell function [32,33]. This defect in beta-cell compensation seems to result from the interaction between environmental factors and genetic predisposition. Shu *et al.* [34] reported that regulation of TCF7L2 might play a critical role in the regulation of both beta-cell survival and function and that targeting its expression could be a new strategy to maintain beta-cell survival in T2DM. In addition, Florez *et al.* [7] suggested that common variants in TCF7L2 were associated with an increased risk of diabetes in persons with impaired glucose tolerance and supported the notion that the risk-conferring genotypes in TCF7L2 were associated with impaired beta-cell function but not with insulin resistance. TCF7L2 is expressed in most human tissues, including mature pancreatic beta-cell, with the exception of the skeletal muscle [8]. Interestingly, TCF7L2 expression in adipose tissue is decreased in obese subjects with T2DM [8]. The TCF7L2 gene encodes a transcription factor of the canonical Wnt signaling pathway, which is one of the key development and growth regulatory mechanisms of the cell. Wnt signaling plays a substantial role in beta-cell proliferation and insulin secretion [35,36] and influences synthesis of glucagon-like peptide 1 (GLP-1) in intestinal cells [37]. The GLP-1, in concert with insulin, plays an important role in blood glucose homeostasis, and it has been postulated that TCF7L2 gene variants may influence the susceptibility to T2DM by indirectly altering GLP-1 levels [37]. TCF7L2 has been identified in glucose homeostasis through the regulation of pro-glucagon gene expression, which encodes GLP-1 in intestinal cells [34]. Furthermore, Lyssenko *et al.* [38] illustrated that the increased risk of T2DM conferred by variants in TCF7L2 involves the enteroinsular axis, enhanced expression of the gene in islets, and impaired insulin secretion. To date, the precise mechanism of action of TCF7L2 in glucose metabolism and the pathogenesis of T2DM has yet to be determined, but it is possible that TCF7L2 has a role in regulating glucose-sensitive insulin secretion from beta-cell.

The discovery of genetic variants of the TCF7L2 gene contributing to genetic susceptibility to T2DM is a breakthrough in the genetic study of T2DM. Research about the association between genetic polymorphisms of TCF7L2 gene and T2DM was first reported by Grant *et al.* [6], they demonstrated that a strong association of variants in TCF7L2 with increased risk of T2DM in Icelandic individuals, and this association was replicated in Danish and US cohorts (combined *OR* = 1.56, *p* = 4.7×10^−18^). Since the original publication, numerous reported studies have examined this gene, with consistent replication in different ethnic populations. In fact, the TCF7L2 gene is regarded as one of the most influential genes in determining the genetic susceptibility for T2DM in human beings. van Vliet-Ostaptchouk *et al.* [9] reported that variants of the TCF7L2 gene might affect the susceptibility toT2DM in a Dutch population and found that the minor allele of the rs12255372 was significantly more prevalent in T2DM patients than control subjects (34% *vs.* 29%, *p* = 0.003), and the frequencies of the heterozygous genotype (GT) and homozygous genotype (TT) were also increased in T2DM patients (*p* = 0.012). Kimber *et al.* [10] found that TCF7L2 was an important gene for determining susceptibility to T2DM in a large case-control study in UK and suggested that variants of TCF7L2 might be associated with increased T2DM severity and therapeutic failure. Gonzalez-Sanchez *et al.* [11] found that the risk allele frequencies of rs7901695, rs7903146 and rs12255372 were significantly higher in T2DM patients compared with that in control subjects and suggested that genetic variant in the TCF7L2 gene were major genetic contribution of the risk of T2DM in Spain. Lehman *et al.* [12] confirmed that variations of theTCF7L2 gene might confer the risk of T2DM in Mexican Americans, but the attributable risk might be much lower than in Caucasian population. Sale *et al.* [13] indicated that variants in the TCF7L2 gene might contribute to genetic susceptibility to T2DM in African-American populations. In the study of Hayashi *et al.* [14], all investigated polymorphisms (rs12255372, rs7903146, rs7901695 and rs11196205) were significantly associated with T2DM in Japanese populations, and rs12255372 revealed the strongest association (*OR* = 1.7, 95% confidence interval (*CI*): 1.20–2.41, *p* = 0.0024), although the frequency of the minor allele in Japanese population was substantially lower than that in European populations. Chandak *et al.* [15] indicated that genetic variation in TCF7L2 gene might be significantly associated with T2DM in India, as previously reported in European populations. In those studies, the frequency of risk allele of rs12255372 was showed to be more frequent in non-Asian populations (MAF = 0.217–0.267), suggesting that the genetic background of Asians is different from that of other ethnic populations, such as European, American, African, and others.

In our study, we found that polymorphisms of TCF7L2 were associated with risk of T2DM in the Uygur population. There was a significant difference in the genotype distribution of rs12255372 and rs7901695 between T2DM patients and control subjects. For total participants, the T allele frequency of rs12255372 was higher in T2DM patients than in control subjects. When male and female were analyzed separately, for male, the T allele frequency of rs12255372 was higher in T2DM patients than in control subjects. There was no difference of T allele between T2DM patients and control subjects in female. This result indicated that T allele of rs12255372 was a risk factor for T2DM in male patients. For total participants and male, the dominant model (GG *vs.* GT + TT) was significantly higher in controls than in subjects with T2DM. For male, the recessive model (TT *vs.* GG + GT) was significantly higher in subjects with T2DM than in controls, after multivariate adjustment of confounding factors, such as serum concentration of TG, TC, HDL, and LDL for T2DM, the significant difference was retained. This result indicated that the risk of T2DM was increased with the presence of the TT genotype of rs12255372 in Uygur male. For total participants, the C allele frequency of rs7901695 was higher in T2DM patients than in control subjects. When male and female were analyzed separately, for male, the C allele frequency of rs7901695 was higher in T2DM patients than in control subjects. There was no difference of C allele between T2DM patients and control subjects in female. This result indicated that C allele of rs7901695 was a risk factor for T2DM in male patients. For total participants and male, the dominant model (TT *vs.* TC + CC) was significantly higher in controls than in subjects with T2DM, the recessive model (CC *vs.* TT + TC) was significantly higher in subjects with T2DM than in controls, after multivariate adjustment of confounding factors such as serum concentration of TG, TC, HDL, and LDL for T2DM, the significant difference was retained. This result indicated that the risk of T2DM was increased with the presence of the CC genotype of rs7901695 in Uygur male. In addition, haplotypes were constructed on the basis of the genotype data from two representative common SNPs using Haploview software. The two SNPs, rs12255372 and rs7901695, were found to be in strong linkage disequilibrium with each other (*D’* = 0.879, *r^2^* = 0.671) and therefore formed a haplotype block. For total participants and male, individuals carrying the CT haplotype, composed of both two risk alleles, showed a significantly increased risk of T2DM (total: *p* = 0.006; male: *p* < 0.001), whereas individuals carrying the wild-type haplotype TG showed a significantly decreased risk of T2DM (both *p* = 0.002). This result is consistent with that which showed that both the C allele of rs7901695 and the T allele of rs12255372 were significantly associated with increased risk of T2DM, as both the C allele of rs7901695 and the T allele of rs12255372 were involved in the CT haplotype. For female, the CG haplotype, which carries the C allele of rs7901695 was significantly associated with increased risk of T2DM (*p* = 0.044). 

In the present study, when male and female were analyzed separately, the distribution of rs12255372 and rs7901695 genotypes showed a significant difference between T2DM patients and control subjects in male. No such differences were observed among female. This result might be caused by two reasons. Firstly, the sample size of Uygur female was relatively small, which limited the statistical power to detect an association between TCF7L2 and T2DM in female. Secondly, it might be because of estrogen. Estrogen plays a very important role in many physiological and pathological processes, such as lipid metabolism, glucose metabolism and insulin related signal transduction pathways [39]. Several previous studies indicated that TCF7L2 plays a key role in glucose homeostasis. Therefore, estrogen, directly or indirectly, affects the function of TCF7L2 gene.

The genetic polymorphisms of the TCF7L2 gene have been reported in epidemiology studies of Chinese Han population, but the association between the TCF7L2 gene and T2DM in Chinese Han population is still controversial. Ren *et al.* [16] found that there was no association between TCF7L2 polymorphism and T2DM risk in Chinese Han population. In the study of Ng, *et al.* [17], another SNP rs11196218, which located in adjacent LD block in the intron 4 of TCF7L2 was significantly associated with increased risk of T2DM in Chinese Han population (*OR* = 1.43, 95% confidence interval (*CI*): 1.14–1.79). Chang *et al.* [18] confirmed that a novel association of the genetic variant SNP rs290487 in the TCF7L2 gene with T2DM in the Chinese Han population. However, they did not found any association of the previously reported risk allele (rs7903146 T and rs12255372 T) with T2DM, which may due to their low frequency in the Chinese Han population. These data revealed that genetic polymorphisms of the TCF7L2 gene were major determinants of T2DM in the Chinese Han population. In our study, the frequency of T allele of rs12255372 was significantly higher in the Chinese Uygur population (MAF = 0.162) than in the Hap–Map Chinese Han population and the Taiwan Chinese Han population (MAF = 0.0040). In studies carried out in the Chinese Han population, the association of rs12255372 with T2DM was not replicated in the Beijing Chinese Han population [16] and Taiwan Chinese Han population [18]. In the study of the Hong Kong Chinese Han population [17] rs12255372 was not tested. These discrepancies can be attributed to the complex of the composition of the Chinese population and the great differences among ethnic populations in genetics and living environment.

There were several limitations in the present study. Firstly, the present study was limited by the relatively small sample size of Uygur female. This may have led to weak statistical significance and wide *CIs* when estimating *ORs*. A larger sample size case–control study is required to investigate the association between the polymorphisms of the TCF7L2 and T2DM in female of the Uygur population. Secondly, additional studies need to be undertaken to clarify the underlying molecular mechanism that associates the TCF7L2 polymorphisms with T2DM.

## 5. Conclusions

In conclusion, this is the first study to investigate the differences between the TCF7L2 gene and T2DM in a Uygur population of China. The present data indicated that the genetic polymorphisms of the TCF7L2 gene were associated with T2DM in a Uygur population of China. This result may broaden the knowledge of genetic variants and disease-association studies.
